# Vitamin D Level in Laboratory Confirmed COVID-19 and Disease Progression

**DOI:** 10.5152/eurasianjmed.2022.21088

**Published:** 2022-10-01

**Authors:** Nasim Dana, Maryam Nasirian, Golnaz Vaseghi, Kiyan Heshmat-Ghahdarijani, Behrooz Ataei, Azam Mosayebi, Amirreza Manteghinejad, Shaghayegh Haghjooy Javanmard

**Affiliations:** 1 Applied Physiology Research Center, Cardiovascular Research Institute, Isfahan University of Medical Sciences, Isfahan, Iran; 2 Infectious Diseases and Tropical Medicine Research Center; and Epidemiology and Biostatistics Department, Health School, Isfahan University of Medical Sciences, Isfahan, Iran; 3 Isfahan Cardiovascular Research Center, Cardiovascular Research Institute, Isfahan University of Medical Sciences, Isfahan, Iran; 4 Heart Failure Research Center, ardiovascular Research Institute, Isfahan University of Medical Sciences, Isfahan, Iran; 5 Infectious Diseases and Tropical Medicine Research Center, Isfahan University of Medical Sciences, Isfahan, Iran; 6 Cancer Prevention Research Center, Isfahan University of Medical Sciences, Omid Hospital, Isfahan, Iran

**Keywords:** COVID-19, Coronavirus, vitamin D, mortality, hospital duration

## Abstract

**Objective:**

There is no conclusive evidence to suggest vitamin D level can prevent or treat infection with the new coronavirus disease 2019. This study aimed to investigate the effects of serum level of vitamin D in patients with coronavirus disease 2019 on death, severity, and hospitalization duration.

**Materials and Methods:**

Baseline characteristic of patients was extracted from the Isfahan coronavirus disease 2019 registry database (I-CORE). Blood samples were taken from all patients to measure the level of vitamin D (25-hydroxyvitamin D) and categorized. The effect of 25(OH) D on death, severity, and hospitalization duration was analyzed by logistic regression.

**Results:**

Among our study patients, 5.5% had a severe deficiency of vitamin D, 23.7% deficiency, and 24.8% insufficiency. Of the 107 patients who died, 7.5% were severely deficient in vitamin D. We found that vitamin D deficiency had no significant effect on death, disease severity, and hospitalization (*P* > .05). However, having at least one comorbidity increased the odds of death five times after adjusting age > 60 years and gender (*P* < .0001). The results showed that among all comorbidities, diabetes has the greatest impact on the outcomes as it raised the odds of death, disease severity, and length of hospital stay by 2.23,1.72, and 1.48, respectively, after controlling the age > 60 and gender (*P *= .0002, *P* = .08, *P* = .012).

**Conclusions:**

The mortality, disease severity, and hospitalization of coronavirus disease 2019 patients seem to be not affected by the low levels of 25(OH)D. However, the synergy between vitamin D levels and comorbidities, age, and gender could affect the outcome of coronavirus disease 2019 patients.

**Main Points**

- No beneficial effect of vitamin D on coronavirus disease 2019 (COVID-19) severity and mortality was observed.
- After adjusting the effect of comorbidities, age, and gender, a significant positive association between vitamin D deficiency and death was seen.
- In the studies which assess the effect of vitamin D on COVID-19, the type of result analysis is one of the factors that influence outcomes and leads to contradictory results in these studies.

## Introduction

Coronavirus disease (COVID-19) is lethal pneumonia caused by novel severe acute respiratory syndrome coronavirus 2 (SARS-CoV-2) and may also be associated with acute respiratory distress syndrome (ARDS).^1^

Although most of the infected patients have some mild-moderate symptoms such as fever and irritation of the respiratory tract, a substantial number of people, mainly in patients with the underlying medical disease, can lead to multiple organ failure and eventual death.^2^

Previous evidence presenting the protective effects of vitamin D suggests the potential impact of vitamin D on SARS-CoV-2 infection.^3^ Vitamin D is a natural immunoregulator with antiviral activity against respiratory viruses.^4^ Several studies reported that vitamin D deficiency influences the risk of respiratory distress syndrome.^5^ Vitamin D amounts can affect surviving patients with ARDS and its deficiency may increase the risk of ARDS. Also, its deficiency can affect lung maturation, structure, function, and volume.^6^

Vitamin D metabolites potentiate antiviral innate immune response, including antimicrobial peptides production and induction of autophagy.^7^ Furthermore, vitamin D receptor is expressed on immune cells has different immunomodulatory and anti-inflammatory effects.^8^ So, it could have a role both in the early stages of the viral infection and the acute inflammatory phase of COVID-19.

Vitamin D deficiency is recognized as an important global health problem and is common in many countries, including Iran.^9^

Some of researchers have examined the beneficial effect of serum 25(OH)D concentrations on various aspects of COVID-19 disease which is proposed to be based on its effect on inflammatory possess and cytokine storm.^10^ On the other hand, some studies did not approve the beneficial impact of this vitamin on COVID-19 outcomes.^11^

Since there is considerable controversy among researchers about the effect of vitamin D in the prevention and progression of COVID-19, we aimed to evaluate vitamin D levels in COVID-19 patients at the time of infection and investigate its relationship with mortality, disease severity, and the length of hospital stay.

## Materials and Methods

### Participants

**Figure 1.**
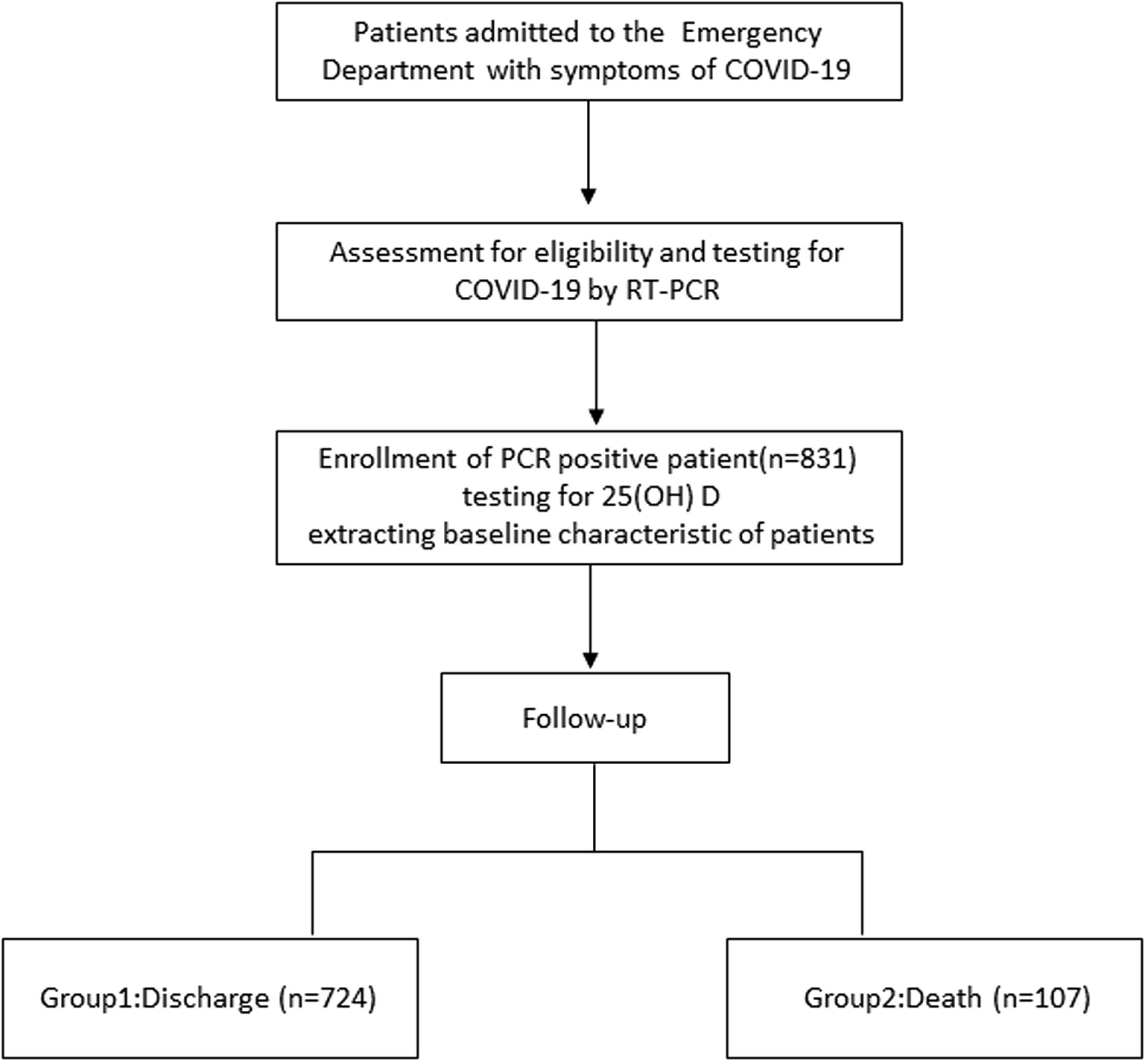
Selection of study population. Flowchart depicting the recruitment of the study patients.

A cross-sectional study was conducted on 831 COVID-19 patients with positive reverse transcription-polymerase chain reaction (RT-PCR) assay results (Figure 1). Patients were entered into the study using convenience sampling among those admitted between March 2020 and November 2020 in hospitals. This research was confirmed by Isfahan University of Medical Sciences (IR.MUI.MED.REC.1398.709). All of the participants were asked to sign an informed consent form before the start of the study.

### Measurements

The baseline characteristic of patients was extracted from the COVID-19 registry database(I-CORE).^12^ At the beginning of the study, blood samples were taken from all patients to measure the amount of 25-hydroxy vitamin D (25 (OH) D) and categorized into: Severe deficiency (0-10), Deficiency (1-20), Insufficiency (20-30), Sufficiency (30-100), Toxicity (upper than 100).^13^ For each patient, age, gender, medical history, lengths of stay in the hospital, comorbid disorder, and mortality were collected as well by review of medical records. Besides, disease severity was defined as Intensive Care Unit admission with oxygen saturation <93. Considering three outcomes, patients were compared in three different groups including alive or dead, severe or non-severe, and more or less than 5 days for hospitalization length.

### Statistical Analysis

Mean (standard deviation [SD]) and median (interquartile range) were used to describe continuous variables as well as frequency and percentage to categorical. The effect of vitamin D on death, severity, and hospitalization duration was examined by binary logistic regression and estimated crude and adjusted odds ratio and 95% confidence interval were reported. Due to the possibility of having several underlying diseases at the same time in patients, we avoided entering all underlying diseases into the multivariate analysis simultaneously. All data were analyzed in Stata Crop software (Version11) considering .05 of significant level.

## Results

### Patient Characteristics

The mean (SD) age of patients was 63.9 (16.2) years and 54.3 percent of them were men. All patients had positive RT-PCR results. Among 831 patients, 724 were discharged alive, and 107 have died. About 74.3% of patients had at least one comorbid disorder; diabetes mellitus (32.6 %) and coronary vascular disease (21.7%) were the most common underlying disease. The Mean hospitalization duration was about 8 days among 724 alive patients. However, 48.5% of them were hospitalized for more than 5 days (Table 1).

The mean (SD) of 25(OH) D in patients was 31.7 (21.3) ng/ml, and 45.3 % had a Sufficient level. However, 5.5% of patients were in severe deficiency level (<10 ng/ml) of vitamin D, of whom 7.5% died (Table 2).

After controlling gender and having at least one disorder, age over 60 years increased the odds of death, and disease severity about 3 and 1.7 times respectively (*P* < .0001; *P *= .014). The results showed that among all underlying diseases, diabetes has the greatest impact on the outcomes as it raised the odds of death, disease severity, and length of hospital stay by 2.23,1.72, and 1.48, respectively after controlling the age >60 and gender (*P* = .0002, *P* = .08, *P* = .012). Although coronary vascular disease influenced death and hospitalization duration, its effect was not significant after controlling age>60 and gender (*P* > .05), but it increased the probability of disease severity by 68% (*P* = .012). In addition, hypertension increased the chance of death by 60% (*P* = .018). Gender, 25(OH) D < 50, 25(OH) D < 12.5, cancer, chronic kidney disease, chronic respiratory disease, and asthma did not affect any of the outcomes (*P *> .05). However, having at least one underlying disease increased the odds of death five times after adjusting age >60 years and gender (*P* < .0001) (Table 3).

The results showed that after adjusting the effect of having at least one underlying disease, the odds of mortality in women with vitamin D deficiency was about five times higher than in women with normal serum vitamin D (*P* = .039). Also, women with 25(OH) D>100 ng/ml were ten times more likely to be severe disease compared to women with normal amounts of this vitamin (*P* = .014) (Table 4).

## Discussion

Vitamin D agonist, calcitriol, played a protective role in acute lung diseases, supporting the hypothesis that its deficiency may act as a pathogenic agent in novel coronavirus illnesses.^14^ The fact that immunoregulatory effects of vitamin D are mediated via regulation of the renin-angiotensin system has certain significance in the context of severe COVID-19.^15^ It has been assumed that vitamin D plays this role by a different mechanism such as reducing the production of pro-inﬂammatory cytokines, induction of antimicrobial peptides, and improving physical barriers.^16^ Also, it has been shown that in vitro culture of type 2 alveolar cells with vitamin D can increase the activity of surfactant-associated protein B.^17^ This demonstrates its ability to reduce surface tension in COVID-19. Despite these beneficial effects of vitamin D, its role in COVID-19 severity and mortality is still debatable. So in this research, we assessed the influence of vitamin D level in the laboratory-confirmed COVID-19 patients on disease severity and outcome. We observed that the prevalence of sufficient amounts of 25(OH) D among dead patients from COVID-19 was 45.8 % and in severe patients was 40.7%. Our result didn’t show the significant effect of vitamin D insufficiency on death, disease severity, or more than 5 days of hospitalization.

In line with our findings, Tehrani and his colleagues measured vitamin D status in 205 Iranian patients with COVID-19. They observed that there is no significant difference between the mean levels of vitamin D in patients who recovered and those who died.^18^ Similarly; Hastie et al^19^ revealed that low vitamin D status is not linked with COVID-19 progression and death.

In Italy, a group of researchers founded that there are low levels of 25(OH) D in their COVID-19 patients, but no association was found with inflammation markers, clinical severity, or length of hospitalization.^20^ Cereda et al^21^ reported that vitamin D deficiency is not related to COVID-19 signs and symptoms. But, when they adjusted their results for confounding variables, a positive relationship between vitamin D levels and death was observed.

Moreover, the results of some trials could not address the beneficial effect of vitamin D_3_ consumption in COVID-19 patients.^22^

In contrast to the results of our study, some other studies have shown that vitamin D deficiency can influence the course of the disease of patients with COVID-19 in hospital.^11^

Angelidi et al^23^ in a retrospective study on 144 COVID-19 patients, observed that vitamin D_3_ status is inversely related to death in hospital and the need for using a ventilator. In the other research on 186 hospitalized COVID-19 patients, 59% of patients had vitamin D deficiency when they were admitted to the hospital and it was associated with mortality.^24^ Also, Carpagnano et al^25^ after assessment of forty-two patients with acute respiratory failure because of COVID-19 found that patients with hypovitaminosis D had significantly higher mortality risk. Katz et al^26^ observed that low vitamin D levels significantly can increase the risk of COVID-19. It is noteworthy that in this study, patients with a history of vitamin D deficiency (n=87) were enrolled and this vitamin was not measured at the beginning of the study.

In one research in Korea, investigators after comparing COVID-19 patients (n = 50) with the control group (people which were tested for vitamin D one year before the study) observed that vitamin D was significantly lower in COVID-19 patients in comparison with normal individuals.^27^ In this regard, Abrishami et al^28^ in their research on 73 patients observed that the mean level of 25(OH) D in the dead patients was significantly less than in discharged patients. Also, some other studies suggest a link between low vitamin D levels and a higher risk of COVID-19 infection and hospitalization.^29,30^

It could be assumed that different types of analysis and the impact of not adjusting comorbidities may lead to conflicting results between different studies. So, we adjusted the effect of having at least one underlying disease and then analyzed the results. Interestingly our results showed that the odds of death in women with deficient serum vitamin D was about five times higher than in women with sufficient serum vitamin D. Previously it has been shown that vitamin D3 has sex-related immunomodulatory effects and has a crucial role in the inhibition of the cytokine storm.^31^ It has been shown that vitamin D controls the differentiation of T regulatory cells in an estrogen-dependent manner.^32^ Besides, estrogen can affect different subtypes of T cells and alters B cell development and activity. On the other hand, the cause of different effects of vitamin D on men and women may be is due to the effect of endogenous testosterone.^33^ Identification of mechanisms by which gender plays these effects needs more research.

Our results also displayed that among all underlying diseases, diabetes has the greatest impact on the outcomes as it raised the odds of death, disease severity, and length of hospital stay after controlling the age>60 and gender. Although Coronary Vascular Disease influenced death and hospitalization duration, its effect was not significant after adjusting for age>60 and gender but it increased the probability of disease severity by 68%.

Consistent with our results, a meta-analysis showed that comorbidities and the elderly have coloration with vitamin D levels and COVID-19 infection, and adjusting for comorbidity variables and vitamin D status, indicated that diabetes and male gender increased the risk of COVID-19 severity.^34^ It has been reported both elderly and comorbidities such as diabetes, coronary heart disease, hypertension, and also COVID-19 are linked to vitamin D insufficiency.^35^ This suggests that comorbidities in combination with vitamin D deficiency might negatively affect the risk of severe COVID-19 progression.

The authors acknowledge some limitations. One of the limitations of our study is that vitamin D levels may be affected by medications or supplements taken by the patient before hospitalization. We cannot interpret whether low 25(OH)D status on the first day of hospitalization is a cause or a consequence of COVID-19 infection. Although our sample size has enough power for determining differences in the clinical outcomes of COVID19 patients, it may have low power to detect differences if stratified further according to confounding factors. Our results must be interpreted carefully in the context of mild COVID-19 because hospitalized patients with more severe disease courses were included in this study.

In conclusion, although several studies have been performed about the relation between vitamin D and COVID-19 severity and mortality, there is still no agreement about the role of vitamin D. This seems to be due to different designs of studies, differences in the number of patients, and their classification, heterogeneity of the patient populations and failure to adjustment for comorbidities, different cut-off points for serum 25-OHD level sufficiency, time of vitamin D measurement that all may cause conflicting results. So, rigorously designed, adequately powered, randomized, controlled studies are needed to investigate the effect of vitamin D on COVID-19 infection.

## Figures and Tables

**Table 1.** Baseline Characteristics of the Study Subjects with COVID-19

**Characteristics**	**N = 831**
**Age, years**	
Median (IQR)	64 (53-77)
Mean (SD)	63.9 (16.2)
**Gender, n (%)**	
Female	380 (45.7)
Male	451 (54.3)
**Coexisting disorder, n (%)**	
At least one disorder	617 (74.3)
Cancer	25 (3.0)
Asthma	12 (1.4)
Chorionic respiratory diseases	66 (7.9)
Diabetes mellitus	271 (32.6)
Coronary vascular disease	180 (21.7)
Hypertension	325 (39.1)
Chorionic kidney disease	80 (9.6)
Other chronic diseases	177 (21.3)
**Dead patients**	**107 (12.9)**
Severe patients*	**145 (17.5)**
**Hospitalization duration ^&^**	
Mean (SD), days	7.9 (7.3)
Median (IQR), days	5 (9-3)
>5 days, n (%)	351 (48.5)

*Who was admitted to ICU considering O_2sat_ < 93.

&Days between admission to discharge (just for alive patients; n = 724). IQR: interquartile range, ICU, intensive care unit, SD, standard deviation.

**Table 2.** Vitamin D Distribution Based on Different Outcomes

**Total (n = 831)**	**Dead (n = 107)**	Severe* (n = 145)	**Hospitalized > 5 days ^&^ (n = 351)**
	**Patients**			
	**Vitamin D (ng/mL)**				
Median	28.3	28.3	27.3	28.3
(IQR)	(41.1-17.6)	(40.9-18.1)	(39.6-18.9)	(42.8-17.4)
Mean (SD)	31.7 (21.3)	31.6 (21.4)	31.4 (19.3)	32.0 (24.8)
**Vitamin D Level ^#^ , n (%)**				
Severe deficiency	46 (5.5)	8 (7.5)	7 (4.8)	23 (6.6)
Deficiency	197 (23.7)	30 (28.0)	35 (24.1)	73 (20.8)
Insufficiency	206 (24.8)	18 (16.8)	41 (28.2)	93 (26.5)
Sufficiency	376 (45.3)	49 (45.8)	59 (40.7)	159 (45.3)
Toxicity	6 (0.72)	2 (1.9)	3 (2.1)	3 (0.85)

#Severe deficiency (0-10), deficiency (1-20), insufficiency (20-30), sufficiency (30-100), and toxicity (upper than 100).

*Who was admitted to ICU considering O_2sat_ < 93.

^&^Days between admission to discharge (just for alive patients; n=724). IQR, interquartile range; ICU, intensive care unit; SD, standard deviation.

**Table 3.** Associated Factor with Hospitalization Duration, Severity, and Death

**Hospitalization Duration>5 Days^&^**		**Severity**		**Death**	
Crude	Adjusted^#^	Crude	Adjusted^#^	Crude	Adjusted^#^
	**Odds Ratio (95% CI)**					
	**Age>60**	1.25 (0.95-1.66)	1.23 (0.92-1.65)	1.73 (1.17-2.55)*	1.66 (1.10-2.48)*	3.80 (2.24- 6.45)*	2.95 (1.72-5.08)*
	**Female**	0.91 (0.69-1.19)	0.87 (0.67-1.16)	0.81 (0.56-1.16)	0.74 (0.52-1.08)	0.96 (0.63-1.44)	0.79 (0.52-1.21)
**DM**	1.50 (1.12-2.01)*	1.48 (1.09-2.08)*	1.82 (1.26-2.63)*	1.72 (1.22-2.63)*	2.48 (1.64-3.75)*	2.23 (1.46-3.47)*
**HTN**	1.14 (0.86-1.51)	1.08 (0.75-1.44)	1.33 (0.92-1.91)	1.18 (0.71-1.62)	2.21 (1.46-3.33)*	1.62 (1.09-2.58)*
**CVD**	1.42 (1.02-1.98)*	1.37 (0.97-1.92)	1.83 (1.23-2.73)*	1.68 (1.12-2.53)*	1.66 (1.05-2.06)*	1.34 (0.84-2.13)
**CKD**	0.90 (0.54-1.44)	0.90 (0.57-1.44)	1.12 (0.62-2.02)	1.12 (0.62-2.03)	0.85 (0.42-1.75)	0.86 (0.46-1.81)
**Cancer**	0.52 (0.22-1.18)	0.53 (0.23-1.23)	0.63 (0.18-2.15)	0.68 (0.20-2.34)	1.72 (0.63-4.69)	1.99 (0.70-5.63)
**CRD**	0.67 (0.40-1.11)	0.65 (0.39-1.08)	1.05 (0.54-2.02)	0.99 (0.51-1.91)	1.07 (0.51-2.23)	0.94 (0.44-1.99)
**Asthma**	1.32 (0.41-4.20)	1.33 (0.42-4.26)	0.94 (0.20-4.36)	0.97 (0.21-4.52)	0.61 (0.08-4.78)	0.67 (0.08-5.41)
**Have at least one underlying disease**	1.19 (0.87-1.63)	1.13 (0.81-1.57)	1.55 (0.99-2.42)	1.36 (0.85-2.17)	6.78 (2.93-15.7)*	5.11 (2.17-11.9)*
**25(OH) D < 50 nmol/l**	1.03 (0.69-1.52)	1.03 (0.68-1.54)	1.01 (0.60-1.70)	1.01 (0.59-1.72)	0.66 (0.39-1.13)	0.70 (0.40-1.23)
**25(OH) D <12.5 nmol/l**	1.13 (0.78-1.65)	1.12 (0.77-1.64)	0.94 (0.57-1.56)	0.92 (0.55-1.52)	1.36 (0.81-2.28)	1.35 (0.70-2.33)

^&^Days between admission to discharge (just for alive patients; n=724).

*Estimated crude or adjusted odds ratio using binary logistic regression was significant if *P* value <.05.

^#^Age>60 and female gender were adjusted for all variables while had at least one underlying disease was adjusted for age>60, female gender, and 25(OH) D.

**Table 4.** The Effect of Vitamin D on the Hospitalization Duration, Severity, and Death

**Hospitalization Duration>5 Days^&^**		**Severity**		**Death**	
Crude	Adjusted^#^	Crude	Adjusted^#^	Crude	Adjusted^#^
			Odds Ratio (95% CI)*					
			**Vitamin D (continuous)**								
					total	1.00 (0.99-1.00)	1.00 (0.99-1.01)	0.99 (0.99-1.01)	0.99 (0.98-1.01)	1.00 (0.99-1.01)	1.00 (0.99-1.01)
Male	1.00 (0.99-1.01)	1.00 (0.99-1.01)			0.99 (0.98-1.00)	0.99 (0.97-1.01)	1.00 (0.98-1.01)	0.99 (0.98-1.01)
Female	1.00 (0.99-1.01)	1.00 (0.99-1.01)			1.00 (0.99-1.02)	1.00 (0.99-1.02)	1.00 (0.99-1.01)	1.00 (0.98-1.01)
**Vitamin D Categories** ^@^								
Total								
	Sufficiency^^^		1	1	1	1	1	1
	Severe deficiency		1.63 (0.76-3.07)	1.66 (0.88-3.13)	0.96 (0.41-2.25)	1.00 (0.42-2.35)	1.40 (0.62-31.8)	1.60 (0.69-3.73)
	Deficiency		0.91 (0.64-1.28)	0.91 (0.64-1.28)	1.16 (0.73-1.83)	1.16 (0.83-1.75)	1.19 (0.73-1.95)	1.22 (0.75-2.02)
	Insufficiency		1.02 (0.72-1.43)	1.03 (0.73-1.44)	1.33 (0.85-2.07)	1.37 (0.88-2.13)	0.63 (0.36-1.12)	0.68 (0.38-1.21)
	Toxicity		4.79 (0.55-41.4)	4.73 (0.54-40.9)	5.37 (1.06-27.3)	5.27 (1.03-26.9)	3.34 (0.59-18.7)	3.22 (0.54-18.9)
Male								
	Sufficiency^^^		1	1	1	1	1	1
	Severe deficiency		1.38 (0.65-2.89)	1.41 (0.67-2.98)	0.86 (0.33-2.31)	0.91 (0.34-2.38)	0.88 (0.31-2.46)	0.91 (0.31-2.62)
	Deficiency		0.98 (0.621.56)	1.01 (0.63-1.01)	0.95 (0.53-1.73)	0.99 (0.54-1.81)	0.89 (0.45-1.67)	0.94 (0.48-1.82)
	Insufficiency		0.98 (0.61-1.57)	1.03 (0.64-1.65)	1.07 (0.59-1.93)	1.15 (0.63-2.09)	0.43 (0.19-1.94)	0.47 (0.21-1.06)
	Toxicity		No data	No data	No data	No data	No data	No data
Female								
	Sufficiency^^^		1	1	1	1	1	1
	Severe deficiency		2.68 (0.64-10.23)	2.55 (0.65-9.93)	0.66 (0.08-5.41)	0.65 (0.08-5.35)	3.15 (0.77-12.8)	4.96 (1.08-22.8)*
	Deficiency		0.77 (0.44-1.32)	0.77 (0.45-1.35)	1.35 (0.65-2.84)	1.36 (0.64-2.86)	1.71 (0.79-3.66)	1.57 (0.72-3.39)
	Insufficiency		1.04 (0.62-1.72)	1.04 (0.62-1.72)	1.64 (0.84-3.20)	1.64 (0.84-3.20)	0.98 (0.43-2.23)	0.98 (0.42-2.24)
	Toxicity		3.96 (0.44-36.05)	3.97 (0.43-36.2	9.99 (1.59-62.61)*	10.02 (1.59-62.73)*	5.61 (0.89-35.4)	5.97 (0.86-41.28)

^@^Severe deficiency (0-10), Deficiency (1-20), Insufficiency (20-30), Sufficiency (30-100), Toxicity (upper than 100).

^Sufficiency category with the highest case number was considered as reference group.

^&^Days between admission to discharge (just for alive patients; n = 724).

^#^Adjusted for have at least one underlying disease.

*Estimated crude or adjusted odds ratio using binary logistic regression was significant if *P* value <.05.
